# A Novel Approach to Non-Invasive Intracranial Pressure Wave Monitoring: A Pilot Healthy Brain Study

**DOI:** 10.3390/s25134042

**Published:** 2025-06-28

**Authors:** Andrius Karaliunas, Laimonas Bartusis, Solventa Krakauskaite, Edvinas Chaleckas, Mantas Deimantavicius, Yasin Hamarat, Vytautas Petkus, Toma Stulge, Vytenis Ratkunas, Guven Celikkaya, Ingrida Januleviciene, Arminas Ragauskas

**Affiliations:** 1 Health Telematics Science Institute, Kaunas University of Technology, LT-51423 Kaunas, Lithuania; andrius.karaliunas@ktu.edu (A.K.); solventa.krakauskaite@ktu.lt (S.K.); edvinas.chaleckas@ktu.lt (E.C.); mantas.deimantavicius@ktu.lt (M.D.); yasin.hamarat@ktu.lt (Y.H.); vytautas.petkus@ktu.lt (V.P.); toma.stulge@gmail.com (T.S.); vytenis.ratkunas@ktu.lt (V.R.); arminas.ragauskas@ktu.lt (A.R.); 2 Laboratory of Heat-Equipment Research and Testing, Lithuanian Energy Institute, LT-44403 Kaunas, Lithuania; 3 Visual Communication Design, Faculty of Art, Design and Architecture, Isik University, TR-34980 Istanbul, Türkiye; guven.celikkaya@holonext.com; 4 Eye Clinic, Lithuanian University of Health Sciences, LT-50161 Kaunas, Lithuania; ingrida.januleviciene@kaunoklinikos.lt

**Keywords:** intracranial pressure waves, non-invasive monitoring, pulse wave morphology, intracranial compliance, healthy volunteer study

## Abstract

Intracranial pressure (ICP) pulse wave morphology, including the ratios of the three characteristic peaks (P1, P2, and P3), offers valuable insights into intracranial dynamics and brain compliance. Traditional invasive methods for ICP pulse wave monitoring pose significant risks, highlighting the need for non-invasive alternatives. This pilot study investigates a novel non-invasive method for monitoring ICP pulse waves through closed eyelids, using a specially designed, liquid-filled, fully passive sensor system named ‘Archimedes 02’. To our knowledge, this is the first technological approach that enables the non-invasive monitoring of ICP pulse waveforms via closed eyelids. This study involved 10 healthy volunteers, aged 26–39 years, who underwent resting-state non-invasive ICP pulse wave monitoring sessions using the ‘Archimedes 02’ device while in the supine position. The recorded signals were processed to extract pulse waves and evaluate their morphological characteristics. The results indicated successful detection of pressure pulse waves, showing the expected three peaks (P1, P2, and P3) in all subjects. The calculated P2/P1 ratios were 0.762 (SD = ±0.229) for the left eye and 0.808 (SD = ±0.310) for the right eye, suggesting normal intracranial compliance across the cohort, despite variations observed in some individuals. Physiological tests—the Valsalva maneuver and the Queckenstedt test, both performed in the supine position—induced statistically significant increases in the P2/P1 and P3/P1 ratios, supporting the notion that non-invasively recorded pressure pulse waves, measured through closed eyelids, reflect intracranial volume and pressure dynamics. Additionally, a transient hypoemic/hyperemic response test performed in the upright position induced signal changes in pressure recordings from the ‘Archimedes 02’ sensor that were consistent with intact cerebral blood flow autoregulation, aligning with established physiological principles. These findings indicate that ICP pulse waves and their dynamic changes can be monitored non-invasively through closed eyelids, offering a potential method for brain monitoring in patients for whom invasive procedures are not feasible.

## 1. Introduction

The intracranial pressure (ICP) pulse waveform and its parameters provide valuable diagnostic information regarding brain conditions. Experimental studies have shown that the amplitude of the ICP pulse wave depends primarily on the mean value and the pulse amplitude of the cerebral blood volume, depending on the pathology [1]. The ICP pulse waveform is characterized not only by its amplitude but also by the presence of three distinct peaks: P1, P2, and P3 [2,3,4,5]. Other parameters include peak appearance time, rise time coefficient, downward coefficient, wave duration, area under the curve, etc. [6,7]. Overall, 24 metrics can be extracted using an algorithm called Morphological Clustering and Analysis of ICP Pulse (MOCAIP) [8,9].

The first peak, P1, arises from the rapid expansion of the walls of the cerebral arteries in response to the systolic rise in arterial blood pressure. This expansion is transmitted to the cerebrospinal fluid (CSF) and other intracranial media and can thus be identified in the pressure signal [10,11]. The second peak, P2, is associated with an increase in arterial intracranial blood volume [10,11]. This volume increase induces pressure changes within the skull; thus, the amplitude of P2 depends on the compliance of the intracranial compartment. Consequently, the ratio between P1 and P2 is considered indicative of intracranial compliance [12]. The third peak, P3, is linked to cerebral venous blood outflow resistance [10,11].

In a healthy brain, P1 is higher than both P2 and P3, although not all of these peaks are always visible in ICP pulse wave monitoring records [11,13]. Changes in these peaks’ ratios can indicate pathological conditions of the brain. For example, a decreased ratio between P1 and P2 indicates a decline in intracranial compliance [14].

Invasive intraventricular catheter systems remain the gold standard for ICP monitoring [15]. However, their high risk-to-benefit ratio limits their use, primarily in patients with severe brain injuries or certain neurological conditions [16]. In addition, the numeric ICP value alone may lack sufficient diagnostic value, whereas monitoring ICP pulse wave morphology could provide a more personalized diagnostic approach [17]. This approach may have utility in a variety of clinical settings where invasive methods are impractical, including in conscious patients seen in fields such as ophthalmology, neurology, sports medicine, and aerospace medicine. Consequently, there is increasing demand for a non-invasive method to monitor ICP pulse waves and evaluate intracraniospinal compliance.

Researchers from the University of Sao Paulo (Brazil) recently developed a non-invasive method to monitor ICP waveforms [18]. This method involves detecting slight variations in skull deformation using a strain gauge sensor (B4C) placed over the skin of the temporal bone [19]. A clinical study involving 72 brain-injured patients showed a positive correlation r = 0.49 (*p* = 0.001) between mean ICP measured using the invasive Neurovent ICP monitoring system with an optic fiber transducer and the P2/P1 ratio assessed using the B4C sensor [20]. B. Dixon et al. developed a non-invasive brain pulse monitor that captures a brain photoplethysmographic (PPG) signal [21]. A pilot clinical study on 12 patients with implanted extra-ventricular drains revealed a significant correlation (r^2^ = 0.66, *p* < 0.001) between invasive ICP measurements and non-invasive ICP predictions calculated using the PPG pulse wave signal. Another group of researchers developed a method that relies on capacitive measurements of the head’s dielectric properties with electrically isolated electrodes on the scalp. The technology is based on the idea that the head’s dielectric properties change during cardiac and respiratory cycles as a result of periodic changes in intracranial blood and cerebrospinal fluid volumes [22]. In total, 18 young healthy volunteers were included in a head-up (HUT) and head-down (HDT) tilt study. Metrics related to cardiovascular action were extracted from capacitive signals, including the peak-to-valley amplitude (AMP). AMP decreased during HUT (0°: 2869 ± 597 (au); +75°: 2307 ± 490 au, *p* = 0.002) and increased during HDT (−30°: 4403 ± 1428 au, *p* < 0.0001) [22]. Sonovum GmbH (Germany) developed an acoustocerebrography (UltraEasy3ACG) system that utilizes transcranial ultrasound to measure attenuation and the time of flight of ultrasonic pulses to detect dynamical changes in the brain media. M. Sauer et al. conducted a pilot clinical study in septic patients using this system. A total of 20 patients were included in two study groups: the septic shock group and control group. Predictive analysis using the UltraEasy3ACG data presented an accuracy of 83.4%, with a specificity of 89.0% and a sensitivity of 75.1% [23].

We developed a liquid-filled, non-invasive, and entirely passive sensor—one that does not transmit ultrasonic, electromagnetic, or other physical signals—for ICP pulse wave monitoring through closed eyelids. This technology, called ‘Archimedes 02’, was recently tested in patients with traumatic brain injury (TBI) and in 63 normal-tension glaucoma (NTG) patients compared with 68 age- and gender-matched control subjects [24,25]. Preliminary findings from the TBI study indicated a high correlation (R = 0.919–0.960) between invasively recorded ICP pulse waves and those simultaneously recorded using ‘Archimedes 02’ during 37 monitoring sessions. The NTG study suggested that monitoring the amplitude of intracranial pressure pulse waves may serve as a potential biomarker for NTG [24,25].

The aim of this pilot study was to detect intracranial pressure pulse waves with three distinct peaks—P1, P2, and P3—using the ‘Archimedes 02’ device in healthy volunteers, and to assess whether the P2/P1 ratio, an indicator of intracranial compliance, corresponds to values expected in a healthy brain. We also conducted a set of standard physiological tests involving changes in intracranial volume, pressure, and compliance to assess whether the proposed monitor can non-invasively detect these changes in a manner consistent with established knowledge of brain physiology.

## 2. Materials and Methods

### 2.1. Anatomical Basis of the Hypothesis

Due to its anatomical connection to the cerebrospinal fluid via the subarachnoid space of the optic nerve, the human eye may provide a non-invasive means of accessing information regarding CSF pressure dynamics [26,27]. Clinical studies suggest that spontaneous retinal venous pulsation is in phase with intracranial pressure and is likely related to the gradient between ICP and intraocular pressure waveforms [28,29,30]. The optic nerve head also features pulsatile deformations [31]. A recent study identified six ophthalmic biomarkers associated with ICP [32]. State-of-the-art analysis has revealed that no prior studies have been conducted examining the spatial movement of the eyeball caused by ICP waves and changes in the subarachnoid space of the optic nerve.

Our idea is based on the anatomical ‘hydraulic pump’ within the subarachnoid space of the optic nerve, caused by its cul-de-sac anatomy, which translates intracranial pressure to the optic nerve head and moves the eyeball in response to changes in ICP(t) [33]. We hypothesize that subtle ICP pulsations and changes, which move the eyeball, can be monitored through the closed eyelid using a highly sensitive pressure sensor and hydrostatic mechanical contact via a non-compressive liquid between the pulsating eyeball and the digital pressure sensor.

### 2.2. System for Intracranial Pressure Wave Monitoring Through Closed Eyelid

**Figure 1:**
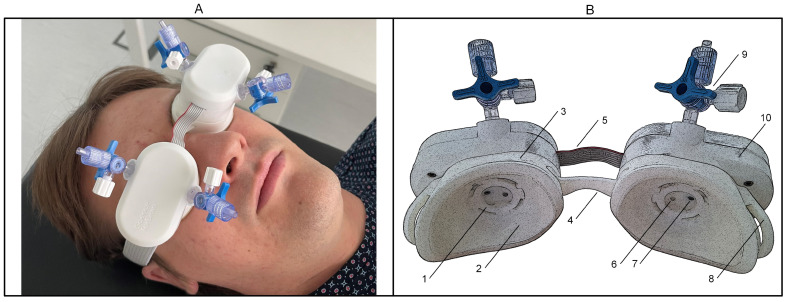
Non-invasive intracranial pressure wave monitor ‘Archimedes 02’. (**A**)—Front view of the device placed on both closed eyelids of a healthy volunteer. (**B**)—Back view of the device, rendered with a poster edge filter to enhance the visualization of the inner parts of the sensor cups: 1—locking mechanism for connecting the sensor module to the disposable cup; 2—transparent elastic film covering the bottom part of the cup; 3—disposable cup; 4—bridge element connecting the right and left cups; 5—signal cable; 6—injection port for liquid; 7—pressure sensor port; 8—buckle for connecting a headband; 9—stopcock for connecting source of liquid; 10—sensor module.

We designed special, non-invasive, wireless, and fully passive sensors that can be placed over both closed eyelids and gently secured with a band around the back of the head (Figure 1). We named the system described below ‘Archimedes 02’. Additionally, we designed a device for single-eye use, intended for cases where only one eye can be used for ICP dynamic monitoring, or where only one needs to be used. This version is called ‘Archimedes 01’.

The two cups that touch the closed left and right eyelids were designed to be disposable, and the sensor modules can be easily connected and disconnected on top of them. The bottom part of the cups is made of thin (50 µm) non-allergic elastic film, which directly touches the closed eyelid. When the sensor modules are hermetically fixed on top of the cups, the inner volume of the cups is filled with a non-compressible liquid. The digital pressure sensors on both the left and right sides are installed so that their stainless-steel pressure ports protrude into the cup, making direct contact with the liquid. The right sensor module contains only a digital pressure sensor, which is connected to the left module’s main control board via a signal cable. Once powered on, ‘Archimedes 02’ can record real-time pressure signals from the left and right sensors separately at a sampling rate of 100 Hz and wirelessly transmit them to a data acquisition application designed for both laptops and smartphones. A Bluetooth low-energy network processor module (BLUENRG-M0L, STMicroelectronics, Plan-les-Ouates, Switzerland) was integrated to manage this communication, utilizing the Bluetooth 5.0 BLE protocol for efficient, low-power data transmission.

### 2.3. Study Protocol

This study was approved by the Kaunas Regional Biomedical Research Ethics Committee (Approval No. BE-2-15, dated 10 February 2024).

A pilot volunteer study was conducted from 18 April 2024 to 4 September 2024 at the Health Telematics Science Institute, Kaunas University of Technology, and the Eye Clinic, Lithuanian University of Health Sciences. Male and female volunteers, aged over 18 years, were included in this study. All subjects signed a written informed consent form to participate in the study in accordance with the Declaration of Helsinki, prior to the study procedures.

Volunteers were placed in the supine position on a tilt table (Teeter Hang Ups Power II Inversion Table, Teeter, Bonney Lake, WA, USA). At first, arterial blood pressure (ABP) was measured with a Microlife (BP B6 Connect, Microlife, Widnau, Switzerland) ABP meter. An automatic triple measurement mode was used for each ABP measurement. Three measurements were automatically taken in succession, and the results were then automatically analyzed and displayed. Then, ‘Archimedes 02’, with both cups filled with liquid, was placed on the volunteer’s closed eyelids. With this setup, the pressure signal was recorded continuously for at least 3 min for all volunteers in the supine position, using our custom-designed software application. Monitoring sessions were extended for some volunteers to compensate for signal artifacts caused by subjects’ movements and to ensure sufficient data collection. After the resting-state monitoring session was completed, subjects who consented were instructed to perform a Valsalva maneuver and a Queckenstedt test; transient hypoemic/hyperemic response tests were also performed.

The Valsalva maneuver is a standardized test in which the subject forcefully exhales against a closed airway, typically by sealing the lips and closing the glottis [34]. During this test, cerebral blood flow is restricted, venous outflow is reduced, and cerebral blood volume increases, leading to elevated intracranial pressure and reduced intracranial compliance [35,36]. In this study, the volunteer, lying in the supine position with the ‘Archimedes 02’ device placed on the closed eyelids, was instructed to exhale and hold their breath; after 10 s, they were instructed to resume normal breathing.

The Queckenstedt test is a standardized clinical procedure in which the jugular veins are manually compressed to temporarily block venous outflow and assess cerebrospinal fluid dynamics [37]. This increases intracranial venous blood volume, thereby elevating intracranial pressure and reducing intracranial compliance [37,38]. In this study, ultrasound-guided compression of the jugular veins was performed for 10 s on a healthy volunteer in the supine position using an ultrasound scanner (EPIQ Elite, Philips, Amsterdam, Netherlands).

The transient hyperemic response test, used to assess dynamic cerebral autoregulation, involves brief compression of the carotid artery while monitoring blood flow velocity in the ipsilateral middle cerebral artery using transcranial Doppler ultrasonography [39,40]. A transient increase in cerebral blood flow velocity following the release of carotid artery compression indicates intact autoregulation, whereas the absence of such a response suggests impaired cerebral autoregulation [39,40]. In our study, ultrasound-guided compression of the common carotid artery was performed for 10 s on a healthy volunteer in an upright position (to minimize the effect of simultaneous jugular vein compression) using a PHILIPS EPIQ Elite ultrasound scanner, while pressure pulse waves from the ipsilateral closed eyelid were monitored using the ‘Archimedes 02’ device.

The ‘Archimedes 02’ device was removed from the volunteer’s head, and ABP was measured again at the end of the procedure.

### 2.4. Data Analysis

Recorded pressure signal data were later processed and analyzed using MATLAB software (version R2024a, MathWorks, Natick, MA, USA). Signal processing consisted of the following six steps:

1. Application of a third-order Butterworth bandpass filter with lower and upper −3 dB cutoff frequencies of 0.5 Hz and 8 Hz, respectively, to remove offsets, slow trends and breathing waves, and to extract pulse waves from the raw pressure signal.
2. Detection of diastolic points in the continuous pressure signal using the standard MATLAB function *findpeaks*, to segment the signal into individual pulse waves.
3. Detrending of each pulse wave individually by subtracting a linear trend defined by the line connecting the first and last points of each pulse wave, bringing the start and end of each waveform to zero.
4. Interpolation or decimation of each pulse wave to standardize its length to 100 data points.
5. Rejection of pulse waves with artifacts to include only valid waveforms in the final calculation of the average pulse wave.
6. Detection of P1, P2, and P3 peaks from the average pulse wave using the standard MATLAB function *findpeaks*. The first detected peak was assigned as P1, the second as P2, and the third as P3 based on their order of appearance in time.

We conducted an analysis to examine the occurrence of three detected peaks in averaged pressure pulse waves collected during resting-state monitoring sessions. The mean ± standard deviation (±SD) values of the P1 amplitude of averaged pulse waves and the ratio of P2/P1 were calculated from the data. Additionally, we calculated the correlation coefficient between averaged pulse waves obtained from the right and left eyes of each volunteer to assess the similarity of signals recorded from both eyes for the same subject.

For the physiological tests performed, pulse wave segmentation was carried out before, during, and after the tests to enable visual inspection of changes in pulse wave shape and in the P2/P1 and P3/P1 peak ratios. These ratios were also calculated for each individual undistorted pulse wave before, during, and after the tests to assess whether they changed significantly during the tests compared to the periods before and after. A two-sided non-parametric Wilcoxon signed-rank test was used to evaluate changes in the P2/P1 and P3/P1 ratios across the three test states. Rank-biserial correlation was used to estimate effect sizes. In addition, the Wilcoxon signed-rank test was applied to compare arterial blood pressure measurements before and after the study procedures. Statistical significance was defined as *p* < 0.05.

## 3. Results

We included 10 volunteers, 5 males and 5 females, in this pilot study. The average age (±SD) was 30.2 (±3.4) years (range: 26–39 years). The characteristics of the individual subjects are presented in Table 1. The mean (±SD) values at the beginning of the experiments were 124.9 (±8.1) mmHg for systolic blood pressure, 71.8 (±3.3) mmHg for diastolic blood pressure, and 69.6 (±15.9) beats/min for heart rate. Although the mean (±SD) systolic blood pressure and heart rate showed some reduction after the experimental procedures—to 121.7 (±10.4) mmHg and 64.9 (±9.9) beats/min, respectively—these changes were not statistically significant (*p* = 0.123 and *p* = 0.109, respectively). Mean diastolic blood pressure showed no significant change (*p* = 0.898), remaining at 71.9 (±4.6) mmHg.

**Figure 2:**
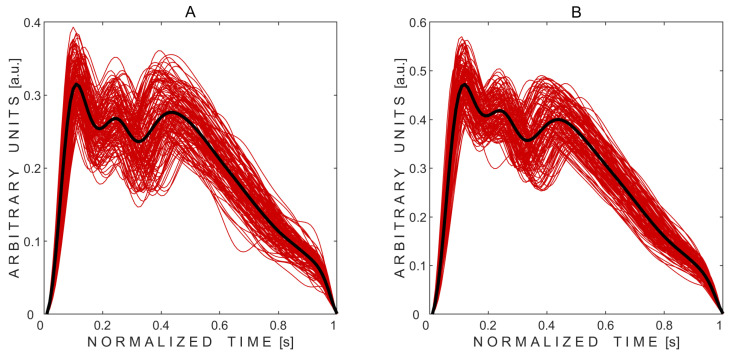
Non-invasively recorded averaged pressure pulse waves (thick black curves) obtained from the left (**A**) and right (**B**) eyes of the subject. Thin red curves represent the recorded pulse waves modulated by physiological intracranial pressure respiratory and slow waves.

After signal processing, pulse waves were successfully extracted from both eyes of all 10 subjects, resulting in 20 averaged pulse waves. Figure 2 demonstrates the recorded valid pulse waves before averaging, along with the averaged pulse waves for the first subject. Distinctly, three peaks can be identified in the recorded pressure waves from both the left and right eyes, with the second peak being lower than the first in both cases.

Each individual pressure pulse wave was standardized to 100 data points to enable averaging across beats. As a result, the time axis was labeled as “normalized time” and scaled to represent a nominal duration of 1 s. Because the signals were filtered and detrended during processing, the resulting amplitudes are expressed in arbitrary units (a.u.).

**Figure 3:**
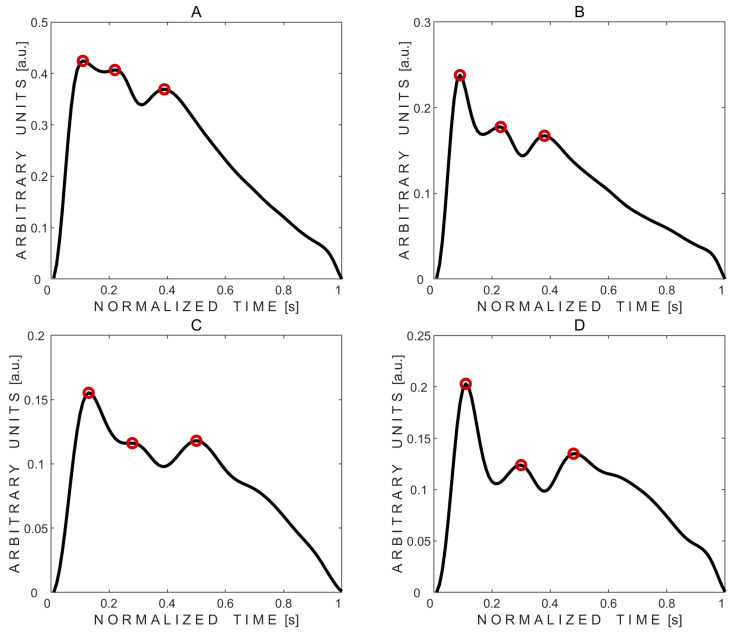
Examples of averaged pressure pulse waves with three automatically detected peaks marked with red circles. (**A**,**B**)—pulse waves recorded from subject No. 2’s left and right eyes, respectively. (**C**,**D**)—pulse waves recorded from subject No. 10’s left and right eyes, respectively.

Three peaks were detected in all 20 average pulse waves. Averaged pulse waves, along with the marked automatically detected three peaks, obtained from volunteers No. 2 and No. 10—who had normal but different intracraniospinal compliances—are shown in Figure 3.

Visual comparison of averaged pulse waves between subjects reveals that subject No. 2 exhibited lower intracranial compliance, as indicated by a higher P2/P1 peak ratio, compared to subject No. 10, despite both displaying physiologically normal waveform shapes.

The results of the resting-state monitoring sessions (without physiological tests) are presented in Table 2, showing the recorded pressure pulse waves for each subject individually. On average, pressure pulse waves were recorded for 3 min and 51 s. The mean (±SD) number of included pulse waves in the final calculations was 210 (±83) and 194 (±66) for the left and right eyes, respectively.

The mean (±SD) P1 amplitude of the averaged pressure pulse waves was 0.217 (±0.103) a.u. for the left eye and 0.189 (±0.104) a.u. for the right eye. On average (±SD), the ratio between peaks P2 and P1 was 0.762 (±0.229) and 0.808 (±0.310) for the left and right eyes, respectively. Both mean values are below 1 and could indicate normal intracranial compliance.

The average (±SD) correlation coefficient R = 0.804 (±0.267) showed a strong relationship between the pressure pulse waves recorded from the left and right eyes.

**Figure 4:**
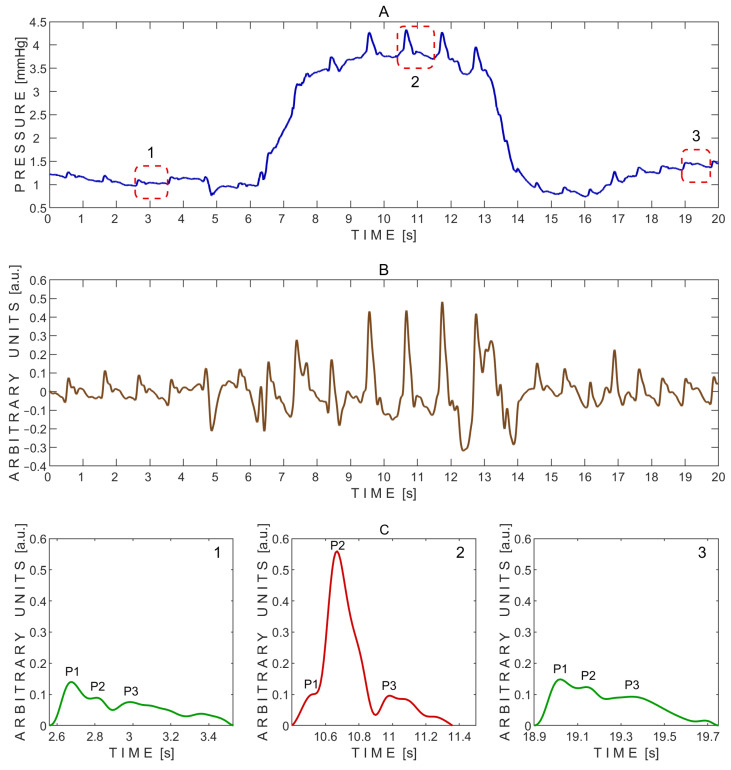
Example of pressure pulse waves recorded from a single subject using the ‘Archimedes 02’ device during the Valsalva maneuver. (**A**)—Raw signal data with three segmented pressure pulse waves indicated by red dashed rectangles: 1—before the Valsalva maneuver; 2—during the maneuver; and 3—during recovery after the maneuver. (**B**)—detrended signal data. (**C**)—Segmented pressure pulse waves extracted from the same subject and corresponding time intervals indicated by red dashed rectangles in (**A**): 1—before the Valsalva maneuver; 2—during the maneuver; and 3—during recovery after the maneuver. P1—first peak; P2—second peak; P3—third peak.

A typical pressure signal recorded non-invasively on a subject through a closed eyelid using the ‘Archimedes 02’ device during the Valsalva maneuver is shown in Figure 4.

The P2/P1 peak ratios were calculated from the exemplary pressure pulse waves extracted before (1), during (2), and after (3) the Valsalva maneuver, as shown in Figure 4C. The corresponding P2/P1 ratios were 0.64 (before), 5.58 (during), and 0.83 (after). Pulse wave 2, recorded during the Valsalva maneuver, showed a markedly elevated P2 peak, resulting in the highest P2/P1 ratio. In addition, the baseline pressure rapidly increased during the Valsalva maneuver from approximately 1 mmHg to 4 mmHg (Figure 4A).

The P2/P1 and P3/P1 ratios were also calculated for each individual undistorted pulse wave. Pairwise comparisons between the three test periods—before vs. during, after vs. during, and before vs. after the Valsalva maneuver—were performed using a two-sided non-parametric Wilcoxon signed-rank test. Statistically significant differences in the P2/P1 ratio were observed between the before and during periods (*p* = 0.016) and between the after and during periods (*p* = 0.016), but not between the before and after periods (*p* = 0.938). The corresponding effect sizes, estimated using the rank-biserial correlation, were 1.000 for both the before vs. during and after vs. during comparisons, indicating large effects. An effect size of 0.071 was observed for the before vs. after comparison, indicating a negligible effect.

Similarly, significant differences in the P3/P1 ratio were found between the before and during periods (*p* = 0.031) and between the after and during periods (*p* = 0.031), while no significant difference was observed between the before and after periods (*p* = 0.938). The effect sizes for these comparisons were both 0.929, reflecting very large effects. An effect size of 0.071 was observed for the before vs. after comparison, indicating a negligible effect.

**Figure 5:**
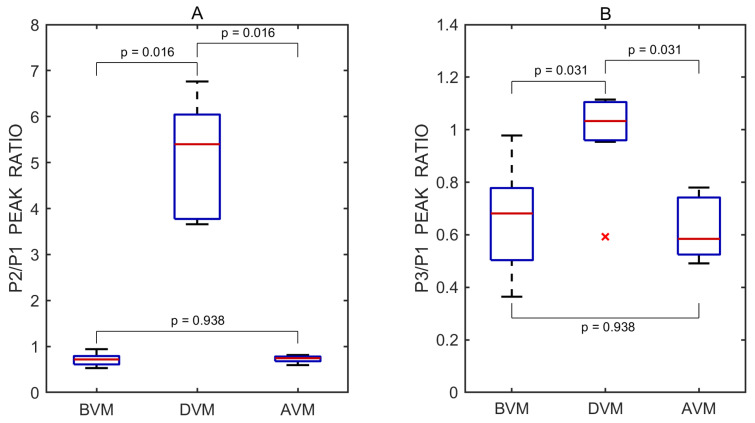
Boxplots of P2/P1 (**A**) and P3/P1 (**B**) peak ratios derived from all recorded pulse wave measurements obtained before (BVM), during (DVM), and after (AVM) the Valsalva maneuver. Red lines indicate medians. A red cross (×) marks a statistical outlier. *p* values represent results from pairwise statistical comparisons.

A total of 21 undistorted pulse waves were included in the statistical analysis, with 7 waves obtained from each test phase (before, during, and after). Boxplots illustrating the effect of the Valsalva maneuver on the P2/P1 and P3/P1 peak ratios are shown in Figure 5.

**Figure 6:**
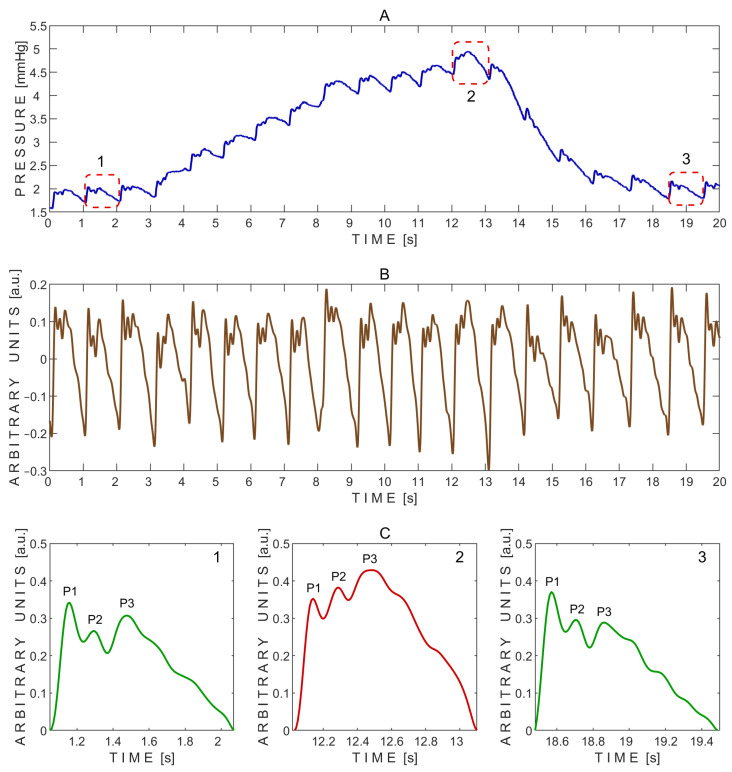
Example of pressure pulse waves recorded from a single subject using the ‘Archimedes 02’ device during the Queckenstedt test. (**A**)—Raw signal data with three segmented pressure pulse waves indicated by red dashed rectangles: 1—before the Queckenstedt test; 2—during the test; and 3—during recovery after the test. (**B**)—Detrended signal data. (**C**)—Segmented pressure pulse waves extracted from the same subject and corresponding time intervals indicated by red dashed rectangles in (**A**): 1—before the Queckenstedt test; 2—during the test; and 3—during recovery after the test. P1—first peak; P2—second peak; P3—third peak.

A typical pressure signal recorded during the Queckenstedt test is shown in Figure 6.

The P2/P1 and P3/P1 peak ratios were calculated from the exemplary pressure pulse waves extracted before (1), during (2), and after (3) the Queckenstedt test, as shown in Figure 6C. The corresponding P2/P1 ratios were 0.78 (before), 1.08 (during), and 0.80 (after), while the P3/P1 ratios were 0.89, 1.22, and 0.78, respectively. In addition, the baseline pressure gradually increased during the Queckenstedt test from approximately 2 mmHg to 4.5 mmHg (Figure 6A).

The P2/P1 and P3/P1 ratios were also calculated for each individual undistorted pulse wave. Pairwise comparisons between the three test periods—before vs. during, after vs. during, and before vs. after the Queckenstedt test—were performed using a two-sided non-parametric Wilcoxon signed-rank test. Statistically significant differences in the P2/P1 ratio were observed between the before and during periods (*p* = 0.027) and between the after and during periods (*p* = 0.004), but not between the before and after periods (*p* = 0.432). The corresponding effect sizes, estimated using the rank-biserial correlation, were 0.782 and 0.964 for the before vs. during and after vs. during comparisons, respectively, both indicating large effects. A small effect size of 0.309 was observed for the before vs. after comparison.

Similarly, significant differences in the P3/P1 ratio were found between the before and during periods (*p* = 0.002) and between the after and during periods (*p* = 0.002), while no significant difference was observed between the before and after periods (*p* = 0.922). The effect sizes for these comparisons were both 1.000, reflecting large effects. An effect size of 0.055 was observed for the before vs. after comparison, indicating a negligible effect.

**Figure 7:**
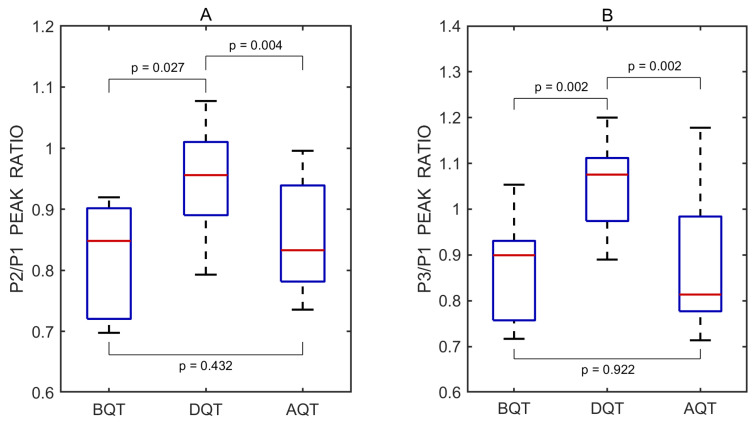
Boxplots of P2/P1 (**A**) and P3/P1 (**B**) peak ratios derived from all recorded pulse wave measurements obtained before (BQT), during (DQT), and after (AQT) the Queckenstedt test. Red lines indicate medians. *p* values represent results from pairwise statistical comparisons.

A total of 30 undistorted pulse waves were included in the statistical analysis, with 10 waves obtained from each Queckenstedt test phase. Boxplots illustrating the effect of the test on the P2/P1 and P3/P1 peak ratios are shown in Figure 7.

**Figure 8:**
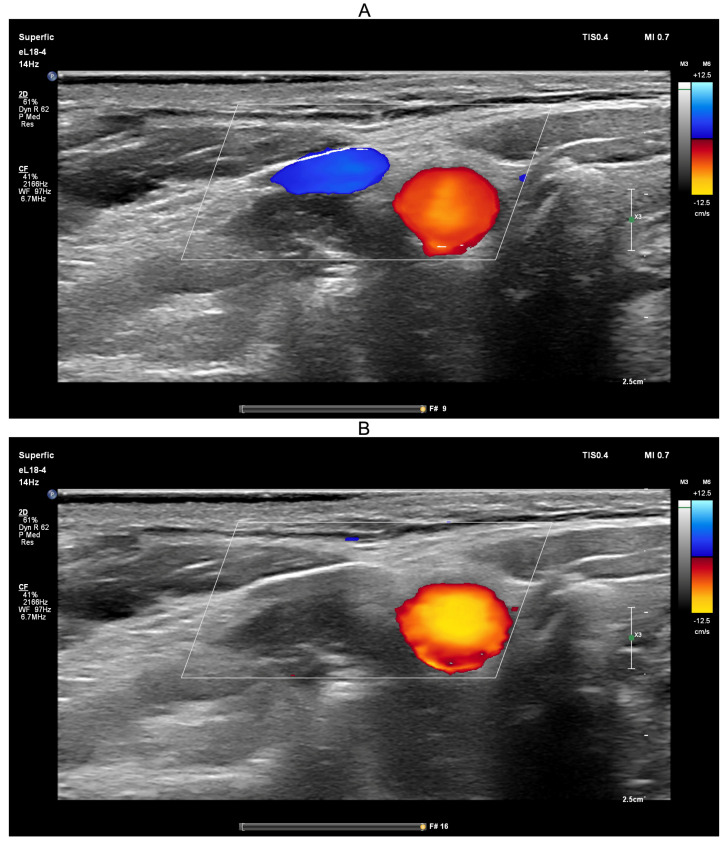
Screenshots from the PHILIPS EPIQ Elite ultrasound triplex scanner during the ultrasound-guided jugular vein compression test (Queckenstedt’s test). (**A**)—Before compression of the jugular veins; (**B**)—During compression of the jugular veins.

Ultrasound-guided compression of both jugular veins was performed on a healthy volunteer in the supine position for approximately 10 s using a PHILIPS EPIQ Elite ultrasound scanner (Figure 8).

Ultrasound guidance was used during the Queckenstedt test to confirm effective bilateral compression of the jugular veins. As shown in Figure 8A, color Doppler ultrasound demonstrated visible jugular venous flow (blue-coded) before compression, which disappeared during compression (Figure 8B), verifying successful occlusion. Simultaneously, carotid arterial flow (red-coded) remained unaffected, confirming selective venous obstruction.

**Figure 9:**
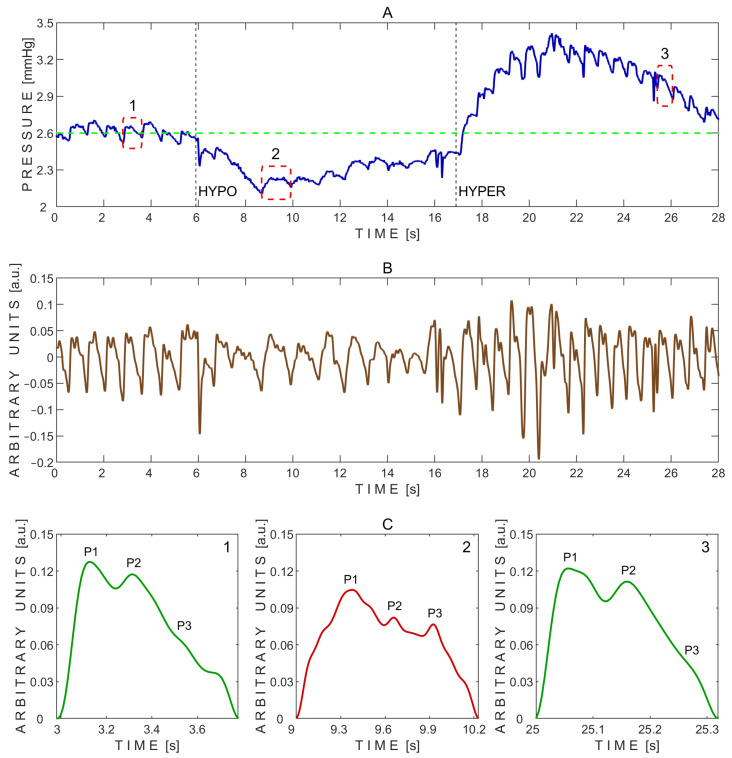
Example of pressure pulse waves recorded from a single subject using the ‘Archimedes 02’ device during the transient hypoemic/hyperemic response test. (**A**)—Raw signal data with three segmented pressure pulse waves indicated by red dashed rectangles: 1—before the test; 2—during the test; and 3—during recovery after the test. HYPO indicates the moment of fast compression of the common carotid artery, and HYPER indicates the moment of rapid release. The green dashed line marks the baseline pressure before the test. (**B**)—Detrended signal data. (**C**)—Segmented pressure pulse waves extracted from the same subject and corresponding time intervals indicated by red dashed rectangles in (**A**): 1—before the test; 2—during the test; and 3—during recovery after the test. P1—first peak; P2—second peak; P3—third peak.

A typical pressure signal recorded during the transient hypoemic/hyperemic response test is shown in Figure 9.

The P2/P1 peak ratios were calculated from the exemplary pressure pulse waves extracted before (1), during (2), and after (3) the transient hypoemic/hyperemic response test, as shown in Figure 9C. The corresponding P2/P1 ratios were 0.92 (before), 0.79 (during), and 0.91 (after). Pulse wave 2, recorded during the test, exhibited the lowest P2/P1 ratio. Additionally, the baseline pressure of 2.6 mmHg dropped to nearly 2.1 mmHg at the onset of common carotid artery compression, followed by a gradual increase toward baseline, reaching approximately 2.4 mmHg (Figure 9A). Upon rapid release of the common carotid artery compression, pressure sharply increased to a peak of about 3.4 mmHg within a few seconds, then gradually declined and settled around 2.8 mmHg (Figure 9A).

The P2/P1 and P3/P1 ratios were also calculated for each individual undistorted pulse wave. Pairwise comparisons between the three test periods—before vs. during, after vs. during, and before vs. after the transient hypoemic/hyperemic response test—were performed using a two-sided non-parametric Wilcoxon signed-rank test. Statistically significant differences in the P2/P1 ratio were observed between the before and during periods (*p* = 0.031) and the after and during periods (*p* = 0.031), but not between the before and after periods (*p* = 0.844). The corresponding effect sizes, estimated using the rank-biserial correlation, were 1.000 for both the before vs. during and after vs. during comparisons, indicating large effects. A small effect size of 0.143 was observed for the before vs. after comparison.

No statistically significant differences in the P3/P1 ratio were found between the before and during periods (*p* = 0.563), the after and during periods (*p* = 1.000), or the before and after periods (*p* = 0.563). The effect sizes for these comparisons were 0.333, 0.048, and 0.333, respectively, all reflecting small effects.

**Figure 10:**
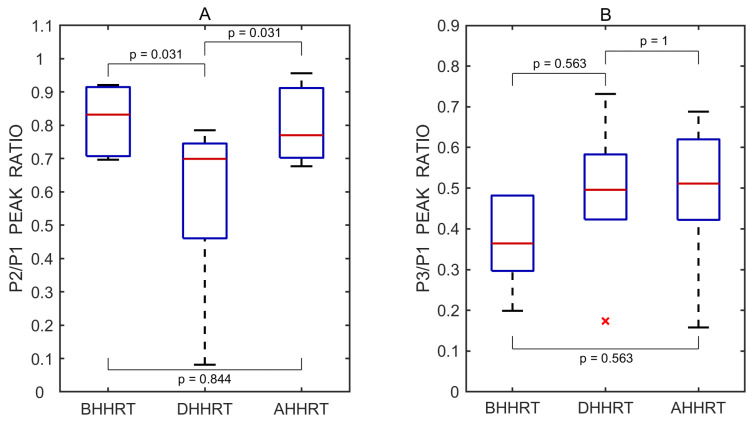
Boxplots of P2/P1 (**A**) and P3/P1 (**B**) peak ratios derived from all recorded pulse wave measurements obtained before (BHHRT), during (DHHRT), and after (AHHRT) the transient hypoemic/hyperemic response test. Red lines indicate medians. A red cross (×) marks a statistical outlier. *p* values represent results from pairwise statistical comparisons.

A total of 18 undistorted pulse waves were included in the statistical analysis, with 6 waves obtained from each test phase (before, during, and after). Boxplots illustrating the effect of the transient hypoemic/hyperemic response test on the P2/P1 and P3/P1 peak ratios are shown in Figure 10.

**Figure 11:**
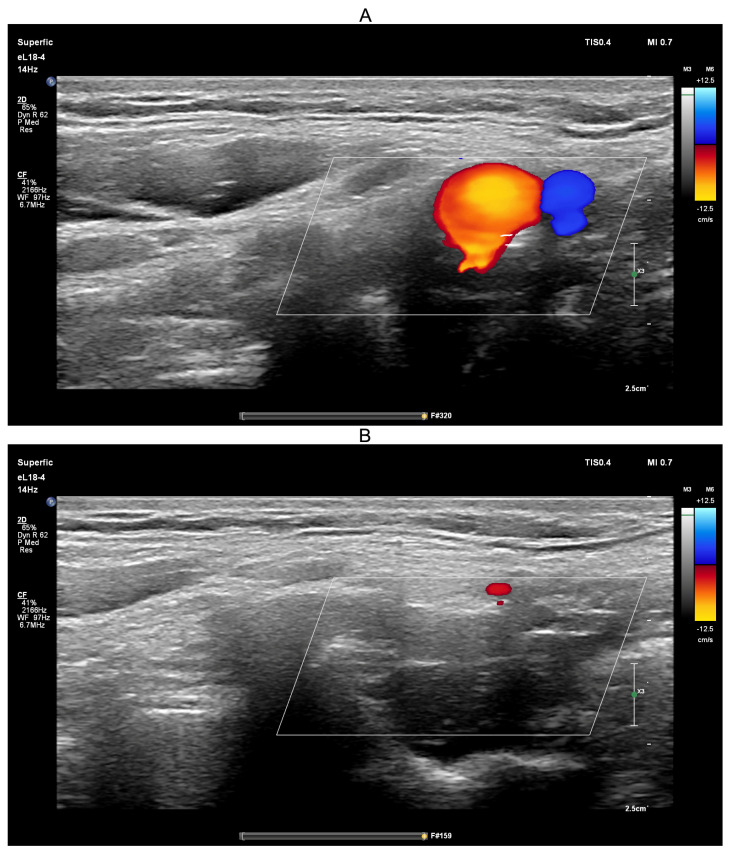
Screenshots from the PHILIPS EPIQ Elite ultrasound triplex scanner during the ultrasound-guided common carotid artery compression test (transient hypoemic/hyperemic response test). (**A**)—Before compression of the common carotid artery; (**B**)—During compression of the common carotid artery.

Ultrasound-guided compression of the common carotid artery was performed on a healthy volunteer in the upright position for approximately 10 s using the same PHILIPS EPIQ Elite ultrasound scanner (Figure 11).

The ultrasound-guided transient hypoemic/hyperemic response test was performed to confirm effective compression of the common carotid artery. Before compression (Figure 11A), red-coded blood flow in the common carotid artery and blue-coded blood flow in the jugular vein were clearly visible. During manual compression (Figure 11B), no color-coded flow was detected, confirming effective arterial occlusion. The procedure was conducted with the participant in the upright position, a physiological state in which jugular venous blood flow is minimal.

## 4. Discussion

The importance of intracranial pressure pulse wave morphology, which provides additional information beyond the absolute ICP value, was recognized decades ago [41,42,43,44]. Intracranial compliance can be reduced after traumatic brain injury, hemorrhagic stroke, and other pathological brain conditions, even when ICP is within the normal range [44]. The ratio between the second and first peaks (P2/P1) extracted from the ICP pulse wave is related to intracranial compliance [12,45]. Although the diagnostic information extracted from the ICP pulse waveform is valuable, currently, it can only be used in intensive care units for patients with implanted invasive ICP sensors. In situations such as diagnosing normal-tension glaucoma, performing cardiac surgery, organ transplantation, or in aero-space medicine, where invasive ICP sensors cannot be implanted into patients’ brains, there is a need for non-invasive monitoring of ICP pulse waves and changes.

Several studies have shown that monitoring skull pulsations, brain photoplethysmographic signals, the dielectric properties of the head, and ultrasonic time-of-flight changes in brain tissue have the potential to estimate characteristics related to intracranial compliance non-invasively [19,21,22,23]. The routine clinical applications of these recent technologies are still unclear. The MRI-based studies analyzed in a review paper indicate that the amplitude of brain tissue pulsations ranges from 0.04 mm to 0.8 mm [46]. Recent MRI 3D models can visualize cardiac-induced CSF and brain tissue pulsations in three dimensions over time [47]. However, MRI technology has limitations when physiological multimodal brain monitoring is used in intensive care units.

In this paper, we present a study conducted in healthy brain that demonstrates the ability to monitor intracranial pressure waves and their dynamic changes—associated with alterations in intracranial volume, pressure, and compliance—through closed eyelids using our non-invasive, fully passive sensor.

In this pilot study involving healthy volunteers, we non-invasively detected pressure pulse waves through closed eyelids, each displaying three characteristic peaks similar to those typically observed in ICP pulse waveforms obtained via invasive monitoring techniques, in all 10 subjects and across all 20 eyelids. On average, the P2/P1 peak ratio was 0.762 for the left eye and 0.808 for the right eye during resting-state monitoring sessions, indicating normal intracranial compliance, as expected in healthy subjects. Although taking measurements from both eyes and calculating a mean value for each healthy subject could potentially improve measurement accuracy, it may also obscure physiologically relevant differences between brain hemispheres—differences that could be significant in patients with stroke or traumatic brain injury [48,49]. Therefore, the non-invasive monitoring of ICP waves from both eyelids could potentially provide not only a global physiological assessment of the brain but also hemisphere-specific information.

In this study involving healthy volunteers, it was expected that intracranial pressure pulse waves monitored non-invasively through closed eyelids would be normally similar in shape between waveforms recorded from the left and right eyes of the same subjects. The overall correlation coefficient calculated across all tested subjects showed a strong association between waveforms obtained from the left and right eyes, R = 0.804. For one subject, we did not find significant correlation as we hypothesized, likely due to the anatomical differences between the eyes.

Physiological tests—Valsalva, Queckenstedt, and transient hypoemic/hyperemic response—were conducted to assess whether pressure waves registered non-invasively through the closed eyelids reflect intracranial dynamics.

The substantial increase in the P2/P1 ratio observed during the Valsalva maneuver suggests a transient reduction in intracranial compliance. This pattern aligns with known physiological effects of the Valsalva maneuver, during which increased intrathoracic pressure can lead to elevated intracranial blood volume and pressure [35,36]. The marked elevation of the P2 peak in pulse wave 2 (Figure 4C) reflects this shift, consistent with increased resistance to cerebrospinal fluid outflow. Furthermore, the observed rise in baseline pressure during the maneuver (Figure 4A) supports the presence of increased ICP in response to the hemodynamic challenge [36].

The increased P2/P1 and P3/P1 ratios observed during the Queckenstedt test suggest reduced intracranial compliance and elevated intracranial pressure, likely due to transient venous outflow obstruction. The elevation of both P2 and P3 peaks in pulse wave may reflect increased intracranial blood volume and cerebral venous congestion, consistent with the known physiological effects of the Queckenstedt test [37,38]. The temporary rise in the P3/P1 ratio may also indicate elevated cerebral venous pressure due to impaired drainage during jugular vein compression. In addition, the gradual increase in baseline pressure from approximately 2 mmHg to 4.5 mmHg during the test (Figure 6A) further supports the presence of elevated intracranial pressure due to restricted jugular outflow [37,38].

During the transient hypoemic/hyperemic response test, the cerebral autoregulation system’s response to common carotid artery compression (maneuver HYPO in Figure 9A) demonstrated an intact autoregulatory mechanism, as indicated by the characteristic rise in pressure pulse waves toward the baseline pressure (green dashed line) following the onset of hypoemia. Similarly, the release of compression (maneuver HYPER in Figure 9A) elicited a typical transient response consistent with cerebral hyperemia, further supporting the presence of intact cerebral blood flow autoregulation [39,40]. The decreased P2/P1 ratio observed at the beginning of common carotid artery compression (0.79), compared to values before (0.92) and after (0.91) compression (Figure 9C), suggests a transient improvement in intracranial compliance and temporary reduction in intracranial pressure, likely due to brief carotid artery inflow obstruction and a subsequent decrease in cerebral arterial blood volume. These findings are consistent with previous studies describing the dynamic behavior of cerebral autoregulation in response to transient changes in cerebral blood flow [39,40].

Taken together, the findings from the Valsalva, Queckenstedt, and transient hypoemic/hyperemic response tests support the interpretation that the recorded pressure pulse waveforms from the closed eyelids are of intracranial origin and reflect underlying intracranial pressure dynamics.

Arterial blood pressure was measured before and after the procedures conducted in this study. To ensure that participants did not exhibit arterial hypertension, ABP was assessed prior to the procedures. All volunteers were considered normotensive, with an average systolic pressure of 124.9 mmHg, diastolic pressure of 71.8 mmHg, and a pulse rate of 69.6 beats per minute. Post-procedural ABP measurements showed no statistically significant changes, indicating that the study interventions were well tolerated and did not induce measurable adverse cardiovascular effects.

Several limitations of our study must be mentioned. First, a limited number of healthy volunteers were enrolled. While the sample size of ten participants is relatively small, it is acceptable for a pilot study targeting the early-stage evaluation of novel technology. At this initial phase, the primary objective was to assess the feasibility, safety, and ability to capture meaningful physiological signals under controlled conditions. Next, we did not implement gold standard ICP pulse wave monitoring for comparison. While invasive intracranial pressure monitoring remains the reference standard, it is only ethically and clinically justified in patients with specific neurological conditions, such as traumatic brain injury or hydrocephalus. Given that our study involved healthy volunteers, the use of invasive monitoring was neither appropriate nor permissible. However, the physiological responses observed using the investigated non-invasive ICP pulse wave sensor are consistent with established principles of cerebral physiology, supporting the plausibility of the recorded signals and their potential relevance for future comparative studies.

## 5. Conclusions

In this pilot study, we observed that it is possible to detect intracranial pulse waves non-invasively through closed eyelids, with three distinct peaks similar to those typically detected in intracranial pressure pulse wave signals obtained through invasive monitoring techniques. This was achieved using a specially designed, liquid-filled, non-invasive, fully passive sensor system incorporating high-resolution pressure sensors. The results obtained during the Valsalva maneuver, the Queckenstedt test, and the transient hypoemic/hyperemic response test support our approach for the non-invasive monitoring of intracranial pressure waves and changes through closed eyelids.

## Figures and Tables

**Table 1:** Characteristics of the volunteers included in this study.

No.	Age, Years	Gender	ABP Before, mmHg			ABP After, mmHg		
			SYS	DIAS	PULSE	SYS	DIAS	PULSE
1	29	Male	122	70	64	132	77	64
2	27	Male	141	69	52	144	72	54
3	26	Female	117	73	51	110	71	53
4	32	Female	128	75	66	120	74	68
5	31	Male	131	70	58	128	71	58
6	29	Male	132	76	107	121	75	86
7	39	Male	125	72	70	122	72	65
8	30	Female	120	77	86	119	78	78
9	30	Female	111	70	67	106	68	59
10	29	Female	122	66	75	115	61	64

Abbreviations: ABP before, arterial blood pressure measured before the experimental procedures; ABP after, arterial blood pressure measured after the experimental procedures; SYS, systolic blood pressure; DIAS, diastolic blood pressure; PULSE, heart rate measured in beats per minute.

**Table 2:** Recorded pressure pulse wave data from resting-state monitoring sessions.

No.	Record Duration	Number of Valid Pulse Waves		Amplitude (P1), a.u.		Ratio of P2/P1		R
		Left	Right	Left	Right	Left	Right	
1	3 min 1 s	131	124	0.315	0.472	0.851	0.887	0.996
2	3 min 12 s	146	125	0.424	0.238	0.958	0.746	0.971
3	3 min 0 s	107	125	0.151	0.122	0.678	0.506	0.724
4	3 min 1 s	173	175	0.084	0.111	0.196	1.122	0.071
5	4 min 3 s	210	197	0.259	0.129	0.843	1.265	0.918
6	4 min 20 s	314	320	0.152	0.130	0.555	0.171	0.650
7	3 min 4 s	176	160	0.331	0.181	0.830	0.717	0.858
8	6 min 24 s	389	288	0.156	0.102	0.997	1.021	0.929
9	5 min 1 s	262	243	0.147	0.197	0.960	1.036	0.979
10	3 min 17 s	194	184	0.155	0.203	0.748	0.611	0.943

Note: Record duration, the time span of the pressure pulse waves’ recording; valid pulse waves, pulse waves included in the calculation of the average pulse wave; a.u., arbitrary units; R, the correlation coefficient between average pulse waves obtained from the left and right eyes.

## Data Availability

The data that supports the findings of this study can be obtained from the corresponding authors upon reasonable request.
